# Screening and Management of Subclinical Hypothyroidism in Pregnancy: A Nationwide Survey of Physicians in Saudi Arabia

**DOI:** 10.7759/cureus.89614

**Published:** 2025-08-08

**Authors:** Raghad Alhejaili, Elsir Mohammed, Beenish Maqsood, Deema Almuhaimel

**Affiliations:** 1 Preventive Medicine, Riyadh Second Health Cluster, Riyadh, SAU; 2 Obstetrics and Gynecology, Women's Health Hospital, Dr. Sulaiman Al Habib Medical Group, Riyadh, SAU; 3 College of Medicine, King Saud University, Riyadh, SAU

**Keywords:** ata guidelines, clinical practice variation, levothyroxine, maternal-fetal outcomes, physician adherence, pregnancy, saudi arabia, subclinical hypothyroidism, tsh threshold

## Abstract

Background

Subclinical hypothyroidism (SCH) in pregnancy poses serious maternal and fetal risks, including miscarriage, gestational diabetes, and neurodevelopmental impairment. Despite clear international guidelines like those from the American Thyroid Association (ATA), global practice remains inconsistent. In Saudi Arabia, where SCH prevalence among pregnant women is notably high (13%), there is limited national data on how closely physicians follow these guidelines. This study aimed to evaluate current clinical practices, adherence to ATA recommendations, and influencing factors across medical specialties.

Methods

A descriptive cross-sectional study was conducted from January to April 2025 using a nationwide online survey targeting licensed physicians involved in obstetric care. The structured questionnaire, adapted from a validated US-based tool, collected data on the SCH screening methods, diagnostic thresholds, use of anti-TPO antibodies, levothyroxine (LT4) prescribing patterns, and guideline familiarity. Adherence scores were computed based on alignment with key ATA recommendations. Statistical analyses, including Chi-square tests, t-tests, and analysis of variance (ANOVA), were performed to assess associations between adherence and physician-related variables.

Results

A total of 280 physicians participated, with most practicing in government hospitals (188, 67.1%) and in the Central Region (220, 78.6%). Although all participants (280, 100.0%) reported screening based on risk factors or symptoms, only 65 (23.2%) had reviewed the ATA guidelines, and 12 (4.3%) applied the recommended TSH cutoff of >2.5 mIU/L in the first trimester. Routine anti-TPO testing was reported by 99 (35.4%) participants, and only nine (3.2%) adhered to the four-week TSH monitoring interval. The mean adherence score was 6.17 (SD = 3.84), significantly associated with physician confidence (r = 0.310, p < .001) and experience (r = 0.323, p < .001). Endocrinologists and academic physicians demonstrated the highest adherence, while general practitioners and physicians from the Western Region showed the lowest. Clinical decision-making was primarily influenced by TSH levels (32, 11.4%), symptoms (18, 6.4%), and guideline awareness (14, 5.0%). Most respondents (208 (74.3%) prescribed LT4 monotherapy, often initiating fixed low doses.

Conclusion

The study revealed substantial variability and low overall adherence to the ATA guidelines among physicians in Saudi Arabia managing SCH during pregnancy. Gaps were most evident in the TSH cutoff application, antibody testing, and postpartum management. These findings highlight the need for targeted education, national consensus protocols, and specialty-specific training to standardize care and improve maternal-fetal outcomes.

## Introduction

Subclinical hypothyroidism (SCH) can be biochemically characterized as an elevation of the serum thyroid-stimulating hormone (TSH) levels along with normal levels of free thyroxine (FT4) [1]. Although usually asymptomatic, the occurrence of SCH can have notable impacts during pregnancy due to the higher metabolic requirements of gestation. These include the higher need for thyroid hormones, higher thyroid-binding globulin (TBG) concentrations, higher urinary excretion of iodine, and higher metabolism of thyroid hormones by placental enzymes. The above changes are crucial to support fetal growth, in particular neurodevelopment, the need for which greatly depends upon the transplacental transport of thyroxine of the mother during early gestation [2].

Global prevalence of SCH among normal, nonpregnant women of reproductive age has been estimated as ranging from 2% to 3% [3,4]. But more significant prevalence rates have emerged from Saudi Arabia. One study from Riyadh reported 13% prevalence among pregnant women compared to 10.3% among the entire female population presenting to primary healthcare centers in Riyadh [5,6]. The result indicates the possible underrecognition or differential diagnosis of SCH during pregnancy at the local level.

Multiple studies have described the adverse effects of SCH during pregnancy. Compared to pregnant women who are euthyroid, pregnant women with SCH are at increased risk of miscarriage, preterm delivery, gestational diabetes mellitus (GDM), placental abruption, and neonatal complications, including low birth weight and neonatal death [7,8]. Additionally, untreated SCH has been associated with preterm delivery, and studies demonstrate that the early initiation of levothyroxine (LT4) may mitigate some of these risks [7].

Despite the availability of international and national guidelines, such as those from the American Thyroid Association (ATA) and the Saudi Society of Endocrinology and Metabolism (SSEM), clinical variation persists in how physicians approach screening and management of SCH, especially when compared to overt hypothyroidism [9,10]. These include factors such as disagreement on TSH cutoffs, doubt about the importance of anti-thyroid antibodies, and variable thresholds for treatment as factors contributing to practice.

SCH in pregnancy presents diagnostic and therapeutic challenges worldwide, with variability in medical practice, inconsistent adherence to guidelines, and patient-specific factors affecting outcomes [10]. International literature highlights key knowledge and practice gaps relevant to Saudi Arabia, where this study aims to provide a national estimate.

Global studies consistently report inconsistencies in SCH screening and management. In the US, a 2021 study by Toloza et al. identified education and communication deficiencies regarding LT4 therapy, leading to anxiety and poor adherence [11]. A 2019 US physician survey, also by Toloza, revealed wide variation in management even among those aware of guidelines [12]. German studies stressed the need for accurate LT4 dosing due to its narrow therapeutic index [13]. In Iraq, less than 40% of physicians regularly screened pregnant women or used correct TSH thresholds [14], while Indian studies found specialty-based differences in treatment thresholds and use of anti-TPO testing [15,16]. In Spain, early LT4 initiation significantly reduced miscarriage and fetal loss [17]. Collectively, these findings show persistent practice gaps globally, underscoring the need for national-level assessment in high-prevalence settings such as Saudi Arabia.

Methodologically, most studies used cross-sectional surveys [11,13], while Runkle et al. conducted a prospective intervention [17] and Hamid et al. a systematic review [15]. Patient-focused research, such as Toloza et al. [11], emphasized educational and adherence gaps, whereas provider-focused surveys-including the 2019 US study [12] and work from Iraq and India [14,16]-highlighted inconsistent screening and treatment.

The common theme across studies [11-17] is widespread inconsistency in practice. Despite guideline availability, consensus is lacking on treatment eligibility, initiation timing, and TSH thresholds, with ongoing concerns over inadequate patient education, poor adherence, and specialty-based variation.

With the prevalence of up to 13% of SCH among pregnant women in Riyadh, significantly higher than the global average of 2-3% [5], and the clear risks of undertreatment [7,8], it is highly relevant to understand how physicians practice in the Saudi environment. While international guidelines, such as those by the ATA, offer structured algorithms for LT4 therapy during pregnancy, there is a notable lack of local data on their adoption in Saudi Arabia, representing a significant gap.

Given the high prevalence and the distinct healthcare environment, a customized national study is imperative. This research aims to bridge this gap by being the first comprehensive national review of how physicians in Saudi Arabia perceive, screen for, and treat SCH among pregnant women.

It will also compare adherence patterns between specialties, such as endocrinology, obstetrics, family medicine, internal medicine, and general practice, and examine key determinants of adherence, including the availability of diagnostic tools, physician confidence, and familiarity with current recommendations. By addressing significant research gaps, the study seeks to identify practical strategies to standardize care, reduce practice variation, and improve maternal-fetal outcomes nationwide.

## Materials and methods

This study employed a descriptive cross-sectional design to evaluate the screening and management practices of SCH during pregnancy among physicians in Saudi Arabia. Data collection was conducted over four months (January-April 2025) using a nationwide online survey. Google Forms enabled broad geographic participation across academic, governmental, and private healthcare settings.

The survey link was disseminated via WhatsApp, a widely used professional communication platform among Saudi healthcare workers, to facilitate convenience sampling and maximize engagement. It was shared through WhatsApp groups of relevant medical specialties across all regions of Saudi Arabia, including endocrinology, obstetrics and gynecology, family medicine, and internal medicine. Group administrators and members were encouraged to forward the invitation to colleagues in related specialties to broaden participation. This approach enabled nationwide dissemination while maintaining voluntary participation. Although the sampling was not stratified by region, responses were obtained from physicians across major population centers, providing broad geographic and institutional representation.

Eligible participants were licensed physicians practicing in Saudi Arabia and involved in the clinical care of pregnant women at risk for or diagnosed with thyroid dysfunction, including SCH. Included specialties were endocrinology, obstetrics and gynecology, family medicine, internal medicine, and general practice. Exclusion criteria were physicians not involved in antenatal care, interns, medical students, and those not independently licensed.

Recruitment was voluntary, and participants accessed the survey link directly. To minimize social desirability bias, the survey was anonymous and voluntary and included instructions encouraging candid responses without concern for identification. Data were collected using a self-administered questionnaire adapted from a validated US-based national survey (“Practice Variation in the Care of Subclinical Hypothyroidism During Pregnancy”) [12]. The questionnaire was publicly shared by the original authors in an online repository. Permission to use and adapt it for this study was obtained by contacting the corresponding author via email, who confirmed their approval. Minor contextual modifications were made to reflect the Saudi healthcare setting, while maintaining the original structure and reliability. On average, participants required seven minutes to complete the survey.

The questionnaire comprised 35 structured items divided into seven sections: (1) screening practices and diagnostic thresholds; (2) laboratory investigations, including TSH, FT4, and anti-thyroid peroxidase antibodies (anti-TPO-Abs); (3) case-based clinical scenarios for first- and second-trimester decision-making; (4) perceptions of maternal and fetal benefits of SCH treatment; (5) prescribing behaviors, including initiation and dosing of LT4; (6) follow-up and postpartum care; and (7) familiarity with guidelines, confidence in decision-making, and demographic/professional background.

To quantitatively assess physician adherence to the ATA guidelines, a structured adherence scoring system was developed by the principal investigator and reviewed by two clinical experts to ensure alignment with ATA recommendations and relevance to clinical practice based on 18 relevant items extracted from the questionnaire. These items were grouped into four thematic categories reflecting key clinical decision points: (1) screening and initial assessment (five items), including TSH cutoff selection and routine antibody testing; (2) first trimester case scenarios (five items), evaluating responses to hypothetical patients with varying TSH and TPO statuses; (3) second trimester case scenarios (five items), mirroring similar decision-making challenges later in pregnancy; and (4) monitoring and postpartum management (three items), assessing follow-up intervals, therapeutic goals, and discontinuation protocols.

Each item was assigned one point for alignment with the ATA 2017 guidelines and zero for nonadherence, producing a cumulative adherence score ranging from 0 to 18 for each respondent. Higher scores reflected greater consistency with evidence-based recommendations. This scoring system enabled standardized comparison of adherence levels across specialties, regions, and practice settings.

Sample size was calculated using Raosoft’s online calculator [18], based on the estimated number of physicians practicing in the selected specialties in Saudi Arabia (N = 43,491; Saudi Ministry of Health, 2023) [19], assuming a 5% margin of error, 95% confidence level, and an 80% response distribution. An 80% response distribution was assumed as a conservative estimate to ensure adequate power for detecting differences in adherence patterns, consistent with standard practice in survey-based studies when the true distribution is unknown. The minimum required sample was 243 physicians. Ultimately, 280 complete responses were obtained, enhancing statistical power and enabling subgroup analysis by specialty.

Survey data were exported from Google Forms, cleaned, and formatted in MS Excel (Microsoft Corporation, Redmond, Washington, United States). The data were statistically analyzed using IBM SPSS Statistics for Windows, Version 28 (Released 2021; IBM Corp., Armonk, New York, United States). Descriptive statistics were used to summarize demographic and clinical practice features. Chi-square tests compared categorical variable groups, whereas independent-sample t-tests and one-way analysis of variance (ANOVA) compared groups of mean adherence scores. Statistical significance was defined as p < 0.05. These analyses identified associations between physician characteristics (e.g., specialty, confidence, experience) and reported screening and treatment behaviors.

Ethical approval was obtained from the Institutional Review Board (IRB) at King Fahd Medical City, Riyadh (IRB Log No. 24-698C). The first page of the online questionnaire provided study objectives, eligibility criteria, confidentiality assurances, and voluntary participation details. Informed electronic consent was obtained from all participants before proceeding. All data were anonymized, securely stored in encrypted, password-protected databases accessible only to the research team, with findings reported in aggregate to ensure confidentiality.

## Results

The present cross-sectional study was conducted as part of a nationwide survey targeting physicians across Saudi Arabia who are involved in the care of pregnant women at risk of thyroid dysfunction. A total of 280 physicians participated in the survey, which was distributed online between January and April 2025. The sample exceeded the calculated minimum of 243 participants based on a 95% confidence level and a 5% margin of error, considering a target population of over 43,000 physicians practicing in Saudi Arabia.

Table 1 presents the demographic and professional distribution of the 280 participating physicians involved in the care of pregnant women at risk of SCH in Saudi Arabia. Regarding specialties, the majority were obstetricians (94, 33.6%), followed by family medicine physicians (60, 21.4%) and general practitioners (48, 17.1%). Internal medicine specialists accounted for 30 (10.7%), while endocrinologists comprised a combined 48 (17.1%), with 28 (10.0%) practicing general endocrinology and 20 (7.1%) focusing specifically on thyroid disorders in pregnancy.

**Table 1:** The distribution of the participants according to their demographic and professional characteristics (n = 280)

Variable	Category	Frequency	Percent (%)
Professional specialty	Obstetrics	94	33.6
	Family medicine	60	21.4
	General practice (merged)	48	17.1
	Internal medicine	30	10.7
	Endocrinology-general	28	10.0
	Endocrinology-focused on thyroid disorders in pregnancy	20	7.1
Type of practice	Government	188	67.1
	Private practice	72	25.7
	Academic	19	6.8
	Other	1	0.4
Geographical region	Central Region	220	78.6
	Eastern Region	29	10.4
	Western Region	23	8.2
	Southern Region	4	1.4
	Northern Region	3	1.1
	Other	1	0.4

In terms of practice setting, most participants worked in government hospitals (188, 67.1%), with smaller proportions from private practices (72, 25.7%) and academic institutions (19, 6.8%). Geographically, a dominant majority were based in the Central Region (220, 78.6%), while others came from the Eastern (29, 10.4%), Western (23, 8.2%), Southern (4, 1.4%), and Northern (3, 1.1%) regions, reflecting a strong central representation in the sample.

Table 2 shows considerable variation in physicians’ adherence to the ATA guidelines for the screening and management of SCH during pregnancy in Saudi Arabia. While all respondents (280, 100%) reported screening pregnant women based on symptoms, risk factors, or miscarriage history, as recommended by the ATA, only 65 (23.2%) had reviewed the actual ATA guidelines. Furthermore, just 12 (4.3%) reported using the guideline-recommended TSH cutoff (>2.5 mIU/L) in the first trimester.

**Table 2:** Overall physician adherence to ATA guidelines in screening, management, and monitoring of SCH (n = 280) ATA: American Thyroid Association; SCH: subclinical hypothyroidism; TSH: thyroid-stimulating hormone; TPO: thyroid peroxidase

Section	Item/case scenario	ATA recommendation	Adherent (n, %)	Nonadherent (n, %)
Screening & initial assessment	Screening pregnant women	Symptoms, risk factors, or miscarriage history	280 (100.0%)	0 (0.0%)
	Reviewed ATA guidelines	Yes, ATA 2017	65 (23.2%)	215 (76.8%)
	TSH cutoff used (1st trimester)	Fixed or population-based >2.5 mIU/L	12 (4.3%)	268 (95.7%)
	Measurement of free/total T4	Always or when TSH is above the cutoff	194 (69.3%)	86 (30.7%)
	Measurement of TPO antibodies	When TSH is above cutoff	99 (35.4%)	181 (64.6%)
1st trimester scenarios	Case 11: TSH 4.4/TPO+/neck normal	Start thyroid hormone therapy now	145 (51.8%)	135 (48.2%)
	Case 12: TSH 4.4/TPO-/neck normal	Start thyroid hormone therapy now	54 (19.3%)	226 (80.7%)
	Case 13: TSH 4.4/TPO-/small goiter	Start thyroid hormone therapy now	102 (36.4%)	178 (63.6%)
	Case 14: TSH 3.2/TPO+/neck normal	Start thyroid hormone therapy now	70 (25.0%)	210 (75.0%)
	Case 15: TSH 3.2/TPO-/neck normal	Repeat TSH, not start therapy	216 (77.1%)	64 (22.9%)
2nd trimester scenarios	Case 18: TSH 4.4/TPO+/neck normal	Start thyroid hormone therapy now	154 (55.0%)	126 (45.0%)
	Case 19: TSH 4.4/TPO-/neck normal	Start thyroid hormone therapy now	63 (22.5%)	217 (77.5%)
	Case 20: TSH 4.4/TPO-/goiter	Start thyroid hormone therapy now	118 (42.1%)	162 (57.9%)
	Case 21: TSH 3.2/TPO+/neck normal	Start thyroid hormone therapy now	78 (27.9%)	202 (72.1%)
	Case 22: TSH 3.2/TPO-/neck normal	Repeat TSH, not start therapy	211 (75.4%)	69 (24.6%)
Monitoring & postpartum	TSH monitoring interval (until mid-gestation)	Monitor everyone every 4 weeks	9 (3.2%)	271 (96.8%)
	Treatment goal in pregnancy	TSH <2.5 or lower half of trimester-specific range	126 (45.0%)	154 (55.0%)
	Stopping therapy postpartum	Only if dose <50 mcg levothyroxine daily	25 (8.9%)	255 (91.1%)

Regarding laboratory assessment, 194 (69.3%) measured free or total T4 when TSH was elevated, and only 99 (35.4%) routinely tested for anti-TPO-Abs in suspected cases. In clinical case scenarios, adherence varied: 145 (51.8%) initiated treatment in a high-TSH and TPO-positive case (TSH 4.4/TPO+), while adherence dropped to 54 (19.3%) in the same TSH level with negative antibodies. In borderline scenarios, such as TSH 3.2/TPO+, only 70 (25.0%) correctly started treatment, whereas the majority (216, 77.1%) appropriately opted for follow-up when both TSH and TPO were mildly abnormal.

Second-trimester scenarios followed similar trends, with only 154 (55.0%) initiating treatment in clear positive cases and lower adherence in more ambiguous ones. The weakest performance was noted in monitoring practice: only nine (3.2%) followed the recommended four-week TSH monitoring interval until mid-gestation, and just 25 (8.9%) correctly identified the postpartum discontinuation threshold for LT4 (only if dose <50 mcg) (Table 2).

The analysis of adherence scores to the ATA guidelines among 280 physicians revealed substantial variation, with scores ranging from 1 to 15 and a mean of 6.17 (SD = 3.84), indicating inconsistent clinical application of recommendations. Correlation analysis showed that adherence was significantly associated with both physician confidence (r = 0.310, p < .001) and years of experience (r = 0.323, p < .001), while confidence itself correlated strongly with experience (r = 0.515, p < .001). 

Further, one-way ANOVA demonstrated statistically significant differences across specialties, practice settings, and regions. Endocrinologists, especially those focused on thyroid disorders in pregnancy, had the highest adherence (20; M = 8.25), while general practitioners had the lowest (48; M = 2.53).

Academics showed better adherence (19; M = 8.58) than government physicians (188; M = 5.31), and regional analysis showed highest adherence in the Northern Region (3; M = 9.33), and Eastern Region (29; M = 8.10), with the lowest in the Western Region (23; M = 3.78). These findings underscore the influence of professional training, practice environment, and geographical context on physician compliance with evidence-based guidelines for SCH in pregnancy (Table 3).

**Table 3:** Comparison of overall adherence score across professional and demographic variables (n = 280)

Variable	Category	Mean adherence score	F	p-value
Professional specialty	Endocrinology-focused on thyroid in pregnancy	8.25	12.378	.000
	Endocrinology-general	7.71		
	Family medicine	7.00		
	Obstetrics	6.73		
	Internal medicine	5.73		
	General practice	2.53	12.148	.000
Type of practice	Academic	8.58		
	Private practice	7.85		
	Government	5.31		
Geographical region	Northern Region (e.g., Arar, Tabuk)	9.33	4.271	.001
	Eastern Region (e.g., Dammam, Al Khobar)	8.10		
	Southern Region (e.g., Abha, Jazan)	6.50		
	Central Region (e.g., Riyadh)	6.14		
	Western Region (e.g., Jeddah, Makkah, Madinah)	3.78		

The findings revealed that clinical decisions regarding the treatment of SCH in pregnancy were influenced by multiple overlapping factors. The most frequently cited determinant was the patient’s TSH level (32, 11.4%), followed by clinical symptoms (18, 6.4%) and adherence to guidelines (14, 5.0%), with other considerations such as TPO-Ab status, T4 level, and goiter presence each mentioned by around 11 (4.0%).

Less commonly reported factors included history of miscarriage, gestational age, patient preference, and research evidence. In terms of pharmacologic management, most physicians (208, 74.3%) prescribed LT4 alone, while only a minority used combination therapy or nonstandard treatments. Regarding dose initiation, 134 (47.9%) preferred a fixed low dose (25-50 mcg/day), while others tailored dosing based on weight, TSH level, or used full doses, with 55 (19.6%) applying mixed or unspecified approaches. 

Physicians' perceptions regarding the benefit of initiating thyroid hormone therapy in pregnant women with SCH varied notably between the first and second trimesters. In the first trimester, 84 (30.0%) respondents believed therapy would offer a large benefit to both mother and baby, while 59-60 (21.0%-21.4%) anticipated a small benefit, and 28 (10.0%) expected a very large impact. Fewer physicians considered it to have minimal or no effect.

In the second trimester, perceptions shifted slightly: 92 (32.9%) indicated a “moderate” benefit, while fewer expected a large or very large benefit, particularly for the mother. Skepticism about treatment value also increased as more participants believed benefits were very small or negligible later in pregnancy (Table 4).

**Table 4:** Perceived benefit of starting thyroid hormone therapy in the first and second trimesters for mother and baby (n = 280)

Perceived benefit	Mother-1st trimester	Baby-1st trimester	Mother-2nd trimester	Baby-2nd trimester
Moderate impact (unspecified category)	90 (32.1%)	84 (30.0%)	92 (32.9%)	92 (32.9%)
Large impact (>20% reduction in risk)	84 (30.0%)	76 (27.1%)	58 (20.7%)	59 (21.1%)
Small impact (10-20% reduction in risk)	60 (21.4%)	59 (21.1%)	78 (27.9%)	57 (20.4%)
Very large impact (>40% reduction in risk)	28 (10.0%)	32 (11.4%)	26 (9.3%)	31 (11.1%)
Very small impact (<10% reduction in risk)	11 (3.9%)	21 (7.5%)	20 (7.1%)	31 (11.1%)
No benefit	7 (2.5%)	8 (2.9%)	6 (2.1%)	10 (3.6%)

These trends suggest uncertainty regarding the optimal timing of intervention and highlight ongoing practice gaps. Additionally, key findings from clinical practice patterns showed that although all participants (280, 100%) screened pregnant women appropriately, only 65 (23.2%) had reviewed the ATA guidelines, and merely 12 (4.3%) applied the correct TSH cutoff. Low adherence was also observed in the use of TPO antibody testing (99, 35.4%) and postpartum monitoring (25, 8.9%). Clinical case scenarios further exposed inconsistent adherence, particularly in TPO-negative cases or when TSH levels were borderline. 

## Discussion

This research sought to evaluate the current clinical practice and the adherence to the recommendations of the ATA among physicians in Saudi Arabia for the screening and management of SCH in pregnant women. It also investigated specialty-based differences, the effect of physician awareness and confidence, and potential areas of improvement to achieve the best possible outcomes in pregnant women and the fetus.

These results exhibited significant variation and minimal adherence to guidelines. Only 65 (23.2%) of respondents had accessed the ATA guidelines, and only 12 (4.3%) used the correct first-trimester TSH threshold of >2.5 mIU/L. These low percentages highlight a significant knowledge-behavior gap, in line with results of Qasim et al. in Iraq, where only 36.9% of doctors applied the <2.5 mIU/L threshold and 27.2% followed up TSH during treatment of LT4 [14]. Such low use of guideline-recommended thresholds and follow-up protocols could lead to delayed detection or inappropriate management of SCH, potentially putting patients at risk of adverse outcomes. Laboratory assessment practices also showed gaps: 194 (69.3%) measured free or total T4 when TSH was elevated, and only 99 (35.4%) routinely tested for anti-TPO antibodies in suspected cases, indicating incomplete evaluation.

Low physician adherence to the ATA guidelines is likely influenced by several factors. As noted by Sudhakaran et al. [16], resource limitations, such as the cost or limited availability of TPOAb antibody testing, may discourage routine screening. In a US survey, Toloza et al. [12] reported wide variation in the TSH diagnostic cutoffs used by physicians, with very few adhering to population-specific reference ranges. Such practice variation is further compounded by conflicting recommendations across different guidelines, which a systematic review by Hamid et al. [15] identified as a major source of physician hesitancy and inconsistent care. These barriers are consistent with the variations observed in our study cohort and highlight the need for harmonized, accessible, and well-disseminated clinical protocols.

The consequences of missed or delayed intervention in maternal thyroid dysfunction are well-established. Haddow et al. demonstrated that children born to untreated overt hypothyroid mothers had significantly lower IQ at 7-9 years, with 19% scoring ≤85 versus 5% in controls [20]. Pop et al. found that maternal fT4 levels below the 10th percentile at 12 weeks’ gestation, despite normal TSH, were associated with lower psychomotor scores at 10 months (RR = 5.8; 95% CI: 1.3-12.6), an effect confirmed at one and two years [21,22]. Multiple international studies link SCH in pregnancy to adverse outcomes, including GDM, miscarriage, preterm delivery, low birth weight, hypertensive disorders, impaired fetal growth, and NICU admission [23-39]. Examples include Toulis et al. (Greece) reporting increased GDM risk (OR = 1.55) [23], Maraka et al. (US) finding a twofold higher miscarriage risk (OR = 2.01) [4], and van den Boogaard et al. (Netherlands) showing doubled first-trimester miscarriage risk [27]. Although some studies, such as Furukawa et al. (Japan), reported minimal risks [36], most evidence supports early detection and management to reduce adverse outcomes. Variability in findings may be due to differences in diagnostic criteria, timing, and populations, as seen in Cleary-Goldman et al. [40] and Williams et al. [41]. In contrast, Thompson et al. [42] and Andersen et al. (Denmark) [43] demonstrated significant neurodevelopmental risks, including intellectual disability and autism spectrum disorders, underscoring the importance of timely intervention.

One of the most important secondary results of the study was the significant specialty-based variation in practice. The adherence score was significantly higher among endocrinologists (48; M = 8.25) and academic physicians (19; M = 8.58) than among general practitioners (48; M = 2.53), and significant regional variations were evident, highest among Northern Region physicians (3; M = 9.33), and lowest among Western Region physicians (23; M = 3.78). Comparable interspecialty differences were noted by Sudhakaran et al. in India, where 82.5% of endocrinologists were against the treatment of mildly raised TSH (2.5-4 mIU/L), whereas 62.8% of obstetricians and only 39.3% of general physicians were in favor of the same [16]. These findings showed the importance of standardized training and continuing education, especially for the purpose of reaching non-specialists and primary care physicians.

Doctor’s expertise and confidence were highly correlated with adherence (r = 0.310, p < .001; r = 0.323, p < .001), which suggests that more experienced and confident clinicians tend to follow guidelines more closely, as in the global patterns. Toloza et al. [11] showed that the physician's ineffective education and communication reduced patient understanding and adherence to LT4 therapy. Alarming as it was, only nine (3.2%) of the respondents in the current study followed the ATA recommendation of TSH monitoring every four weeks, and only 25 (8.9%) knew how to stop therapy after postpartum, again highlighting the concerns discussed in Hamid et al.’s systematic review of the international guidelines’ discrepancies [15]. Also, our findings align with those of Toloza et al., who also reported suboptimal adherence to ATA recommendations among US physicians, despite guideline availability. Similar to their conclusions, this study confirms that physician familiarity with guidelines and confidence in management decisions are key factors influencing adherence levels [12].

Practice patterns of pharmacologic management again showed heterogeneity. While 208 (74.3%) doctors treated with LT4 monotherapy, almost half (134, 47.9%) began therapy with fixed low doses of LT4, in preference to titrated schedules. German research highlighted the importance of accurate LT4 dosing due to the narrow therapeutic index of the drug and the necessity of achieving pregnancy-specific TSH goals [13]. Runkle et al. in Spain demonstrated that early initiation of LT4 (prior to nine weeks of gestation) lowered fetal loss appreciably [17]. However, physicians’ belief in the benefit of therapy decreased drastically after the first trimester period, even as evidence exists indicating that untreated SCH beyond early gestation remains harmful [17]. Several studies, including Vrijkotte et al. [34] and Maraka et al. [4], have highlighted the consequences of inadequate LT4 dosing, linking low maternal fT4 to delayed infant development and showing that each 1 mIU/L increase in maternal TSH is associated with a 3.2-point decline in childhood cognitive index. Although some reports found no clear associations with autism or attention-deficit/hyperactivity disorder (ADHD) [44], Andersen et al.’s recent findings of elevated autism risk [43] reinforce the urgency of standardizing care and improving clinician education.

Overall, this study successfully met its objectives by identifying major gaps in guideline adherence, specialty- and region-based practice variation, and the influence of physician confidence and awareness. Comparisons with international studies from Iraq [14], India [16], Germany [13], the United States [11,12], and Spain [17] showed that SCH management during pregnancy remains a global challenge characterized by inconsistent practices. Addressing these discrepancies in Saudi Arabia will require coordinated policy efforts, national protocols, and investment in continuing medical education (CME) to ensure all pregnant women receive evidence-based, standardized thyroid care.

Recommendations**

To enhance SCH management, we recommend the execution of continuing medical education (CME) initiatives targeting guideline dissemination, especially among general practitioners and family doctors. Currently, there are no standardized national CME initiatives in Saudi Arabia dedicated to SCH management in pregnancy; existing educational opportunities are generally embedded within broader programs and may not reach all physicians involved in antenatal care. Developing targeted, accessible CME programs with accreditation incentives could enhance physician confidence, promote consistent application of guideline recommendations, and reduce variability in clinical practice. On a policy level, professional societies and hospitals should incorporate standardized monitoring and screening criteria as part of standard antenatal care, and policymakers should support the establishment of national consensus guidelines customized for local settings. Potential policy actions could include developing national clinical pathways, integrating them into antenatal protocols, harmonizing laboratory reference ranges, and ensuring the consistent availability of diagnostic tests such as thyroid peroxidase antibodies (TPO-Abs). Increased physician awareness and confidence from these measures will enhance uniform, evidence-based practice, leading to improved pregnancy outcomes across the country. Finally, while the present study assessed adherence to the ATA guidelines, future research could evaluate adherence to the Saudi Society for Endocrinology and Metabolism (SSEM) guidelines to inform targeted educational and policy strategies.

Limitations

This study has several limitations. It was based on self-reported practice, which can lead to potential recall bias, and its cross-sectional design prevents the assessment of patient outcomes, temporal trends, or the effect of intervention. The sample, though nationally distributed, was dominated by the Central Region, preventing regional generalizability. The use of convenience sampling via a self-administered online format and WhatsApp-based recruitment may have introduced selection bias, affecting the representativeness of the study sample by overrepresenting physicians who are more technologically inclined, engaged in professional networks, or with a specific interest in thyroid disorders; this may particularly affect the representativeness of older physicians. While anonymity was used to minimize social desirability bias, this factor cannot be fully excluded. Finally, the four-month data collection period may not account for potential seasonal variations in clinical practice patterns or survey response rates, which could influence the representativeness of the findings.

## Conclusions

This national study uncovered significant knowledge gaps, low awareness of essential guidelines, and considerable practice variation in the care of SCH in pregnancy in Saudi Arabia. The highest adherence was among endocrinologists and academic physicians, whereas the lowest was among general practitioners. Although aware of the value of therapy, especially in early pregnancy, most doctors failed to use the advised screening thresholds or monitoring intervals. Closing the gap between evidence-based guidance and practical, real-world clinical practice should lead to improved outcomes in the mother and the fetus.

## Appendices

**Table 5:** Screening practices and diagnostic thresholds

0	Do you participate in the care of pregnant women at risk of or with thyroid dysfunction?					
No		Yes				
1	Which pregnant women do you screen for thyroid dysfunction? (check all that apply)					
All of them		With symptoms of thyroid disease (e.g., fatigue)	With risk factors for thyroid disease (e.g., positive family history, autoimmune disease)	With previous miscarriages or adverse pregnancy outcomes (e.g., preterm delivery)	I don’t screen pregnant women for thyroid dysfunction	Other women (please describe)
2	Have you ever diagnosed/treated a pregnant woman with subclinical hypothyroidism (i.e., asymptomatic or minimally symptomatic with abnormally high thyrotropin and normal thyroxine levels)?					
No (go to question #6)		Yes				
3	How many years have you been taking care of pregnant women with subclinical hypothyroidism?					
____ years						
4	How many pregnant women with subclinical hypothyroidism do you estimate you have cared for in the last 6 months?					
____ women						
5	What percentage of your pregnant patients with subclinical hypothyroidism came to your care referred from another clinician?					
____ %						
6	Have you reviewed the clinical guidelines for the management of subclinical hypothyroidism in pregnancy? (check all that apply)					
No		Yes, those produced by the Endocrine Society (2012)	Yes, those produced by the American College of Obstetrics and Gynecology (2015)	Yes, those produced by the American Thyroid Association (2017)*	Yes, other guidelines (describe):	
7	If "Yes, those produced by the American Thyroid Association (2017)" is selected, please answer the following sub-question: I consider that these new guidelines by the American Thyroid Association (2017) changed my clinical practice					
No		Yes				

**Table 6:** Laboratory investigations TSH: thyroid-stimulating hormone; TPO-Abs: thyroid peroxidase antibodies

8	Which thyrotropin (TSH) cutoff you use for the diagnosis of subclinical hypothyroidism in the 1st trimester of pregnancy?					
	Nonpregnant adult cutoff for your lab (type here if you know it)	Population-based cutoff for your lab (type here if you know it)	Fixed cutoff of TSH>2.5 mIU/L	Fixed cutoff of TSH>4.0 mIU/L	Other (please describe)	I am not sure
9	In your clinical practice when would you measure free/total thyroxine (T4) for pregnant women with suspected (subclinical) hypothyroidism?					
	Never	When TSH is above the limit reported in question 8	Only when TSH>10.0 mIU/L	When TSH> some other value (type here)	Always	
10	In your clinical practice when would you measure anti-thyroid peroxidase antibodies (TPO-Abs) for pregnant women with suspected (subclinical) hypothyroidism?					
	Never	When TSH is above the limit reported in question 8.	Only when TSH>10.0 mIU/L	When TSH> some other value (type here)	Always	

**Table 7:** Case-based clinical scenarios for first- and second-trimester decision-making and perceptions of maternal and fetal benefits of SCH treatment TSH: thyroid-stimulating hormone; TPO-Abs: thyroid peroxidase antibodies

The following questions (11 to 15) address the possible initiation of thyroid hormone replacement therapy in a healthy 29-year-old woman who presents for a prenatal visit at the 8th week of her first pregnancy. She has no known history of thyroid disorder, infertility or previous miscarriage. She currently takes no medication and the pregnancy is going well. She had labs done the day of the prenatal visit. Please choose one option for each of the following scenarios:					
11	TSH: 4.4 mIU/L; free T4: normal limits; TPO-Abs: (+); neck physical exam: normal. What would you do?				
Start thyroid hormone therapy now		Repeat TSH within 1 month and determine whether to treat then	No further evaluation		
12	TSH: 4.4 mIU/L; free T4: normal limits; TPO-Abs: (-); neck physical exam: normal. What would you do?				
Start thyroid hormone therapy now		Repeat TSH within 1 month and determine whether to treat then	No further evaluation		
13	TSH: 4.4 mIU/L; free T4: normal limits; TPO-Abs: (-); neck physical exam: small diffuse goiter. What would you do?				
Start thyroid hormone therapy now		Repeat TSH within 1 month and determine whether to treat then	No further evaluation		
14	TSH: 3.2 mIU/L; free T4: normal limits; TPO-Abs: (+); neck physical exam: normal. What would you do?				
Start thyroid hormone therapy now		Repeat TSH within 1 month and determine whether to treat then	No further evaluation		
15	TSH: 3.2 mIU/L; free T4: normal limits; TPO-Abs: (-); neck physical exam: normal. What would you do?				
Start thyroid hormone therapy now		Repeat TSH within 1 month and determine whether to treat then	No further evaluation		
If the answer is “Start thyroid hormone therapy now” in any of the previous section’s questions (11 to 15), please answer the following questions (16 and 17):					
16	How much benefit do you expect pregnant women will gain from the treatment?				
No benefit		Very small impact, e.g., <10% reduction in the risk of adverse pregnancy outcomes	Small impact, e.g., 10-20% reduction in the risk of adverse pregnancy outcomes	Large impact, e.g., >20% reduction in the risk of adverse pregnancy outcomes	Very large impact, e.g., >40% reduction in the risk of adverse pregnancy outcomes
17	How much benefit do you expect the offspring will gain from the treatment?				
No benefit		Very small impact, e.g., <10% reduction in the risk of adverse health/cognitive outcomes to offspring	Small impact, e.g., 10-20% reduction in the risk of adverse health/cognitive outcomes to offspring	Large impact, e.g., >20% reduction in the risk of adverse health/cognitive outcomes to offspring	Very large impact, e.g., >40% reduction in the risk of adverse health/cognitive outcomes to offspring
The following questions (18 to 22) address the possible initiation of thyroid hormone replacement therapy in a 32-year-old woman who presents for a prenatal visit at the 17th week of her first pregnancy. She has no known history of thyroid disorder, infertility, or previous miscarriage. She currently takes no medication, and the pregnancy is going well. She had labs done the day of the prenatal visit. Please choose one option for each of the following scenarios:					
18	TSH: 4.4 mIU/L; free T4: normal limits; TPO-Abs: (+); neck physical exam: normal. What would you do?				
Start thyroid hormone therapy now		Repeat TSH within 1 month and determine whether to treat then	No further evaluation		
19	TSH: 4.4 mIU/L; free T4: normal limits; TPO-Abs: (-); neck physical exam: normal. What would you do?				
Start thyroid hormone therapy now		Repeat TSH within 1 month and determine whether to treat then	No further evaluation		
20	TSH: 4.4 mIU/L; free T4: normal limits; TPO-Abs: (-); neck physical exam: small diffuse goiter. What would you do?				
Start thyroid hormone therapy now		Repeat TSH within 1 month and determine whether to treat then	No further evaluation		
21	TSH: 3.2 mIU/L; free T4: normal limits; TPO-Abs: (+); neck physical exam: normal. What would you do?				
Start thyroid hormone therapy now		Repeat TSH within 1 month and determine whether to treat then	No further evaluation		
22	TSH: 3.2 mIU/L; free T4: normal limits; TPO-Abs: (-); neck physical exam: normal. What would you do?				
Start thyroid hormone therapy now		Repeat TSH within 1 month and determine whether to treat then	No further evaluation		
If the answer is “Start thyroid hormone therapy now” in any of the previous section’s questions (18 to 22), please answer the following questions (23 and 24):					
23	How much benefit do you expect pregnant women will gain from the treatment?				
No benefit		Very small impact, e.g., <10% reduction in the risk of adverse health/cognitive outcomes to offspring	Small impact, e.g., 10-20% reduction in the risk of adverse health/cognitive outcomes to offspring	Large impact, e.g., >20% reduction in the risk of adverse health/cognitive outcomes to offspring	Very large impact, e.g., >40% reduction in the risk of adverse health/cognitive outcomes to offspring
24	How much benefit do you expect the offspring will gain from the treatment?				
No benefit		Very small impact, e.g., <10% reduction in the risk of adverse health/cognitive outcomes to offspring	Small impact, e.g., 10-20% reduction in the risk of adverse health/cognitive outcomes to offspring	Large impact, e.g., >20% reduction in the risk of adverse health/cognitive outcomes to offspring	Very large impact, e.g., >40% reduction in the risk of adverse health/cognitive outcomes to offspring

**Table 8:** Prescribing behaviors, including initiation and dosing of levothyroxine (LT4) TPO-Abs: thyroid peroxidase antibodies; TSH: thyroid-stimulating hormone

25	What factors do you consider when deciding whether to treat a pregnant woman with subclinical hypothyroidism? (check all that apply)											
Patient’s age		Gestational age	Clinical symptoms	History of miscarriage	Presence of goiter in physical examination	TPO-Abs	TSH level	T4 level	Patient’s preferences	Guidelines recommendations	Research evidence	Medication side effects
26	What medication(s) would you prescribe for subclinical hypothyroidism treatment in pregnant women?											
Levothyroxine (T4) only		Levothyroxine (T4) + liothyronine (T3)	Thyroid extracts, e.g., Armour thyroid	Other (please describe)	No treatment is needed (go to question #31)							
27	What initial dose of levothyroxine would you use for the treatment of subclinical hypothyroidism in pregnancy?											
Fixed small dose (25-50 mcg/day)		Fixed full dose (75-100 mcg/day)	Initial dose based on patient’s weight (please describe)	Initial dose based on patient’s TSH level (please describe)	Other (please describe)							

**Table 9:** Follow-up and postpartum care TSH: thyroid-stimulating hormone

28	Until mid-gestation a levothyroxine-treated pregnant woman’s TSH level should be measured:				
On everyone every ____ weeks		Only if TSH is not appropriate for gestational week at first check after levothyroxine initiation every ____ weeks	Other	Never	
29	What is the treatment goal in pregnant women with subclinical hypothyroidism?				
TSH in the lower half of the trimester-specific reference range		TSH <2.5 mIU/L	TSH <4.0 mIU/L	TSH between normal limits for a non-pregnant adult	Other
30	Which women with history of subclinical hypothyroidism during pregnancy should stop thyroid hormone postpartum?				
All		Only those who used <50 mcg levothyroxine daily	When TSH level postpartum are within normal limits for a non-pregnant adult	Other (please describe)	None

**Table 10:** Familiarity with guidelines, confidence in decision-making, and demographic/professional background

31	Please rate your level of confidence in taking care of a pregnant woman with respect to the following:					
31a	Screening and diagnosis of subclinical hypothyroidism					
Very confident		Somewhat confident	Neither confident nor not confident	Somewhat not confident	Not at all confident	
31b	Treatment and follow-up of subclinical hypothyroidism					
Very confident		Somewhat confident	Neither confident nor not confident	Somewhat not confident	Not at all confident	
31c	Stopping therapy of subclinical hypothyroidism					
Very confident		Somewhat confident	Neither confident nor not confident	Somewhat not confident	Not at all confident	
32	What best describes your specialty?					
Endocrinology, practice is general, not focused on thyroid in pregnancy		Endocrinology, focused on thyroid disorders including thyroid in pregnancy	Obstetrics	Internal medicine	Family medicine	Other, please specify below:
33	How many years have you been in clinical practice?					
____ years						
34	Is your practice considered:					
Academic		Private practice	Government	Other, please specify below:		
35	What is your geographical location?					
Central Region (e.g., Riyadh)		Western Region (e.g., Jeddah, Makkah, Madinah)	Eastern Region (e.g., Dammam, Al Khobar)	Southern Region (e.g., Abha, Jazan)	Northern Region (e.g., Arar, Tabuk)	
